# Imaging Method for Measurements of Particle Density and Solid Holdup of Entangled MWCNTs in a Fluidized Bed

**DOI:** 10.3390/ma12122035

**Published:** 2019-06-25

**Authors:** Min Ji Lee, Sung Won Kim

**Affiliations:** School of Chemical and Material Engineering, Korea National University of Transportation, Chungju-si, Chungbuk 27469, Korea; mj1226@ut.ac.kr

**Keywords:** carbon nanotube, fluidized bed, apparent particle density, imaging method, Hg porosimetry, solid holdup

## Abstract

A measurement method of the apparent particle density of the carbon nanotube (CNT) particles, characterized by enveloped volume formed by loosely entangled nanotubes, has been proposed for the CNT fluidized bed application. The method is characterized by obtaining the enveloped volume from the CNTs imaging under the free falling condition similar to the fluidized bed. The shape of the falling CNT particles in a column (0.1 m long × 0.012 m wide × 0.60 m high) was photographed using a high-speed camera under the sedimentation condition, and the apparent CNT particle density was calculated from the enveloped volume obtained by image-processing for the particles images. The apparent densities and solid holdups by the imaging method at various conditions were compared with those by the previous Hg-porosimetry method for the two types of CNTs (a vertically aligned CNT and two entangle CNTs) and the nonporous polycarbonate particle (a reference particle). The imaging method reflects well the packed bed and fluidized bed phenomena observed in the experiments with reasonable solid holdups, compared with the Hg-porosimetry method showing high densities and low holdups. The sizes of CNT particles predicted with the density by the imaging method were in good agreement with the measured mean particle sizes when calculated based on the Richardson–Zaki equation, indicating the imaging method represented well the enveloped volume and shape formed by entangled nanotubes on the CNTs.

## 1. Introduction

Carbon nanotube (CNT) particles are nano-scale particles that are attracting attention as new materials in the field of nanotechnology, and have been highly anticipated as promising materials in a wide range of fields due to their excellent properties such as high thermal conductivity and electrical conductivity [1]. Various methods such as catalytic chemical vapor deposition (CCVD), laser ablation, and electric arc-discharge have been developed as CNT particle synthesis techniques. [1,2,3]. Among them, the application of CCVD in a fluidized bed reactor is advantageous in large-scale synthesis of the CNTs due to the advantages of the fluidized bed having a large particle mixing ratio and heat and material transfer efficiency as well as a wide reaction surface area utilization on the catalyst [3].

The multi-walled CNTs (MWCNTs) by the CCVD are produced in two forms, entangled CNTs (ENCNTs) and vertically aligned CNTs (VACNTs), depending on the catalyst and operating conditions [4]. The VACNT has the feature that the nanotubes grow into a long bundle at right angles to the surface, and the ENCNT has a shape in which relatively short nanotubes are entangled with each other on the particle surface [5]. These CNT particles are characterized by a strong aggregation phenomenon by the van der Waals attraction in the fluidized bed reactor [6,7,8]. In addition, the shape and length of nanotubes grown on the CNTs affect the degree of physical entanglement between nanotubes or particles, resulting in the formation of different types of aggregates [9]. Therefore, the study on hydrodynamic behavior of the CNT based on particle shape characterization is very important for reactor design and operation optimization [7]. Among various hydrodynamic properties, the axial solid holdup distribution in the fluidized bed is an important factor that can explain the fluidization quality of the particles, the mixing degree of the particles in the reactor, and the reactor efficiency [10]. 

The solid holdup (*ε_s_*) values in the reactor can be obtained from measured pressure drops along the height and can be calculated from Equation (1) using the gas density (*ρ_g_*) and the particle density (*ρ_p_*) values, assuming that the gas acceleration and the wall friction are ignored [11].

(1) $$\varepsilon_{s}=\;\frac{\Delta P}{\Delta L}/\left[\left(\rho_{p}-\rho_{g}\right)g\right]$$

The gas density in Equation (1) can be obtained in the literature, but the density of the porous particles such as the CNT should be measured experimentally. The apparent particle density should be used as the particle density, which is much hydrodynamically correct if the particle behavior in the gas flow field is of interest as in the fluidized bed [12]. The particle volume on the apparent density of porous particle is replaced with the enveloped volume of the particle as if the particle was nonporous [12].

It is very important to measure the solid holdup distribution of the CNTs with gas velocity to investigate the behavior of the CNT particles in the fluidized bed reactor [8]. So far, many studies have been conducted on the synthesis of the CNTs in the fluidized bed and the cause analysis of their hydrodynamic aggregation [13]. However, the information on the average solid holdup in the bed or its axial distribution with operating conditions is relatively sparse. A few studies [3,14] have shown the bulk density information in their reactor for predicting the particle distribution in the reactor. The bulk density represents the product of the solid density and the solid holdup, so it does not provide information only for the solid holdup.

Recently, a few studies [5,15,16] reported the information on the solid holdup values calculated by Equation (1) using the CNT particles density obtained by the Hg-porosimetry method. The Hg-porosimetry method is first to measures the volume of voids other than the particles by filling the mercury in the voids with increasing system pressure in the container containing the particles, and then to calculates the particle density based on the estimated particle volume [12]. This method is suitable when the definition of the hydrodynamic enveloped shape of a single particle such as the Fluid Catalytic Cracking (FCC) catalyst is clear, because the boundary of the particle surface is clear and the pore size is not influenced by external pressure. However, it is difficult to apply this method in the case of particles such as the CNTs in which the enveloped shape of the particles is formed by the entanglement of the nanotubes on the catalyst surface and the size and shape of the pores between the nanotubes cannot easily be defined. Also, the void in the entangled CNTs may not be recognized as an enveloped volume when the intrusion of the Hg at high pressure may collapse the enveloped shape formed by the nanotubes, or the mercury may easily penetrate between the entangled nanotubes [17]. This indicates a low apparent volume or high particle density, and can lead to the problem of deriving an excessively low solid holdup by Equation (1).

Recently, our previous study [18] showed that the apparent volume of the CNTs can be predicted by the imaging method for a type of the CNT, estimating the particle volume from two-dimensional images of particles. However, the study has a problem of photographing the only one side of the particle shape, since the center of mass of the particle is directed to the plane where they are laid. Therefore, a method for measuring the shape and apparent volume of particles in a free flow state such as a fluidized bed is required. Also, tests for various types of CNT particles and comparative verification tests with nonporous particles are required considering a dependence of the enveloped volume on the shape of the nanotubes. 

In this study, a method of measuring the apparent volume of the CNT particles, having interstitial voids formed by loosely entangled nanotubes, has been proposed. It is characterized by measuring the apparent volume from the CNTs imaging under the free falling condition for the fluidized bed application with the CNT particles of various types. The apparent densities and solid holdups by the method under various conditions were compared with the values obtained by the previous Hg-porosimetry method. In order to determine the validity of the imaging method, the method was compared with the specific gravity measurement methods with non-porous plastic particles, and theoretical verification was performed through the Richardson–Zaki relation [19].

## 2. Materials and Methods 

### 2.1. Material

**Figure 1:**
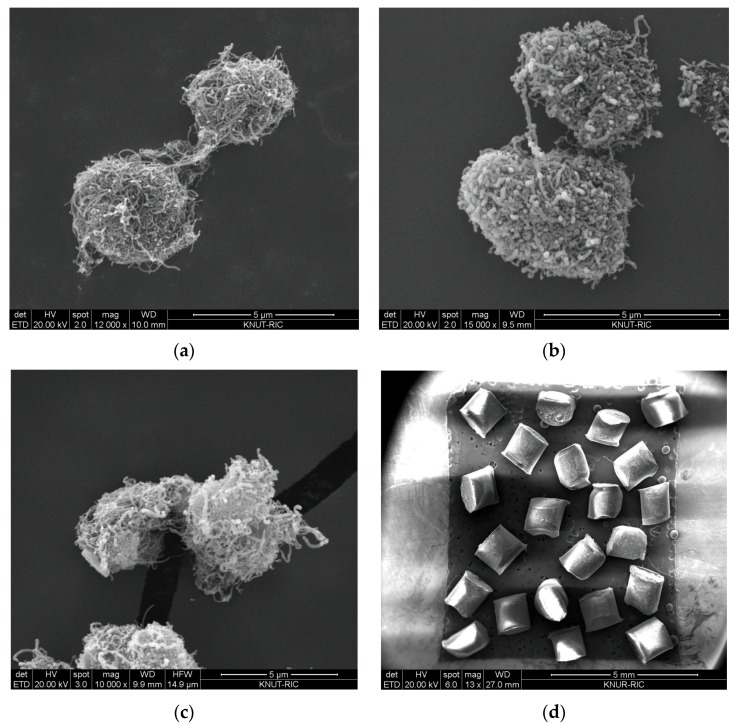
SEM images of particles used in experiments: (**a**) VACNT; (**b**) ENCNT-I; (**c**) ENCNT-II; (**d**) PC (scale bar length: carbon nanotubes (CNTs) = 5 μm; PC = 5 mm).

The CNT particles used in this study are MWCNTs produced in fluidized bed reactors. Two ENCNTs (FloTube™ 9000, C-Nano; NC7000TM, Nanocyl) and a VACNT (FloTube™ 7000, C-Nano) with different nanotube shapes were used as shown in Figure 1. Each ENCNT particle was identified as ENCNT-I and ENCNT-II according to the order indicated above. Nonporous polycarbonate (PC) particles (Maxi-Blast Inc., South bend, IN, US) were used as a reference for the comparative validation of the methodology as in Figure 1d.

Figure 1 shows the images of the scanning electron microscopy (Quanta 400, Bruker, Billerica, MA, USA) to show the typical shape structure of the CNT particles. The VACNT is characterized by intertwined particles by long nanotubes, while the ENCNTs are characterized by a relatively short length of nanotubes, but entangled nanotubes on the same particle. The properties of the MWCNTs and PC particles are shown in Table 1 with mean particle diameter (*d_p_*) and bulk density (*ρ_b_*).

### 2.2. Experimental Apparatus

#### 2.2.1. Sedimentation Column

**Figure 2:**
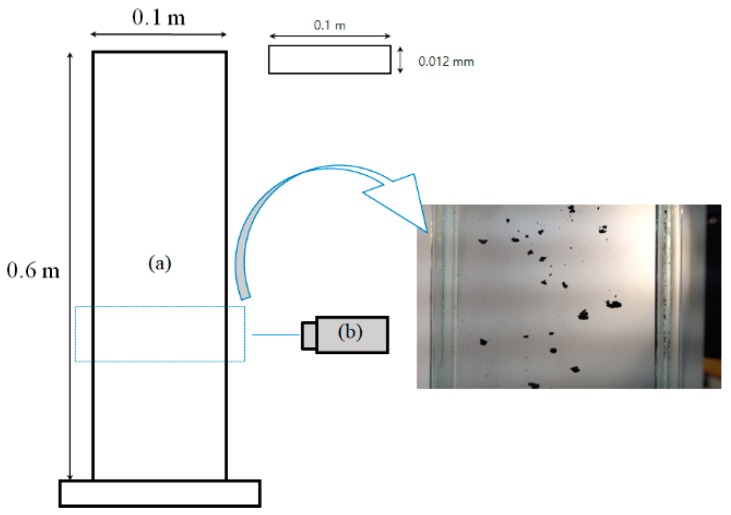
Experimental apparatus: (**a**) Sedimentation column; (**b**) camera.

Experiments were carried out in a sedimentation column (0.1 m long × 0.012 m wide × 0.60 m high) made of transparent glass plate as in Figure 2 to capture the shape of the particles in a free sedimentation condition similar to the flow in a fluidized bed. To capture the shape of individual particles, it is required that the particles settle down within the photographable speed range while preserving the shape without interparticle aggregation during the sedimentation. For this purpose, it is important to select an appropriate solvent as the fluid in which the particles fall down. The ethanol was used as an organic solvent that is hydrophobic and has a large density difference with the MWCNTs to prevent the particle aggregation during the settling [20]. After the ethanol was filled up to 0.4 m-high in the column, the weighed CNT particles were manually scattered and settled down. To capture the shape of falling particles, a high-speed camera (RX100M4, Sony, Tokyo, Japan) was installed at a height of 0.2 m at the opposite side of the column. The amount of CNT particles used in the experiment is 1.0 g, which corresponds to about 140,000 particles in the case of the ENCNT-1.

#### 2.2.2. Fluidized Bed Cold Model Reactor 

**Figure 3:**
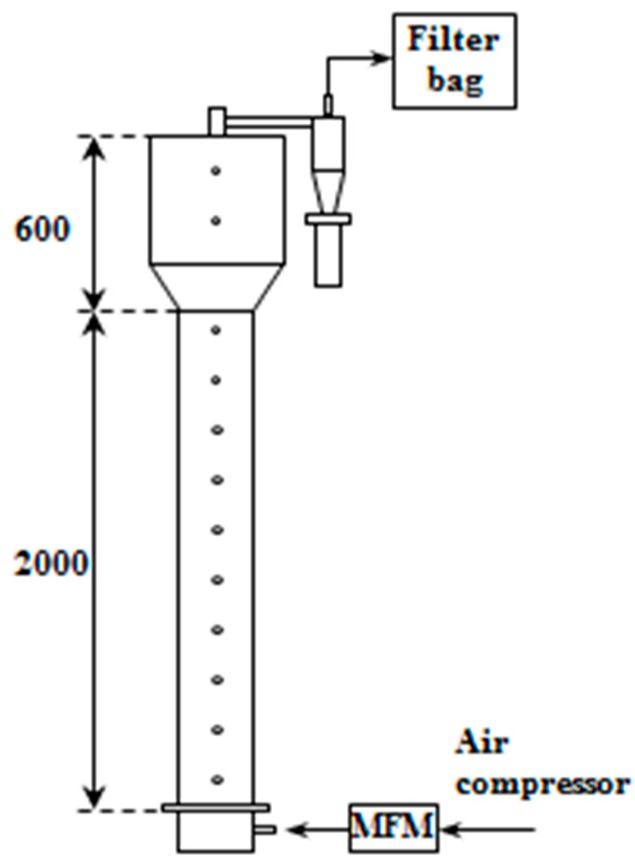
Experimental apparatus for solid holdup measurements.

The solid holdup distributions were measured in a fluidized bed cold model (0.15 m-id and 2.60 m-high) made of transparent Plexiglas column as in Figure 3 to apply the particle densities obtained in the study. The unit consists of a gas distributor of tuyere type and a main column with a cyclone. Air was used as a gas for fluidizing the CNT particles and a mass flowmeter was installed to regulate the air flow rate. Pressure taps were installed at intervals of 0.10 m at the wall of the main column for measurement of the pressure drop across the fluidized bed, which were connected to manometers and pressure transducers. 0.5 kg of the CNT particles as bed materials were filled in the column, which corresponds to a static bed height of about 0.5–0.8 m depending on the bulk density. In the experiment, the gas velocity was varied from 0.06 to 0.20 m/s above the minimum fluidization velocity. The solid holdup values were obtained by Equation (1).

### 2.3. Analysis

#### 2.3.1. Imaging Method

**Figure 4:**
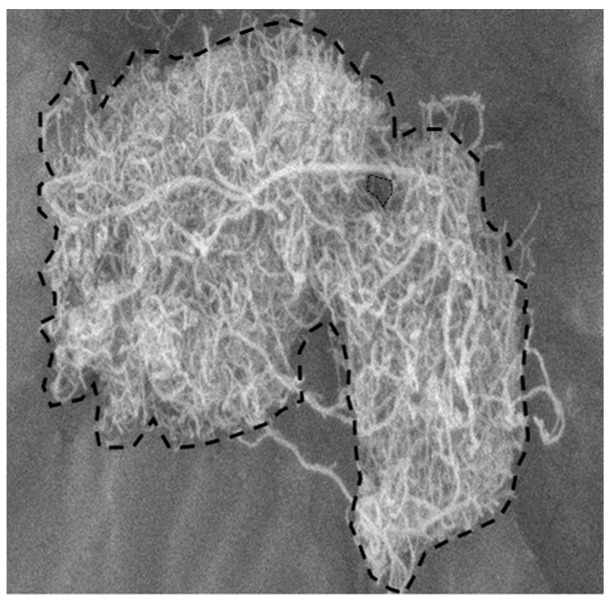
Enveloped boundary of VACNT.

The apparent density of the CNT particles was obtained by measuring the enveloped volume of the individual particles using the imaging method and then dividing the particle weight by the sum of the measured particle volumes. The CNT particle density was measured by a mercury porosimeter (AutoPore V, Micrometrics, Ottawa, ON, Canada) for comparisons with previous studies. The particle density of the nonporous PC particles was measured by a pycnometer for the methodology validation. To obtain the apparent volume of the individual CNT particles, the photographing of the particles settling in the liquid was carried out. The apparent volume was obtained assuming that the CNT particles were spherical based on the equivalent diameter calculated from the enveloped area as in Figure 4.

**Figure 5:**
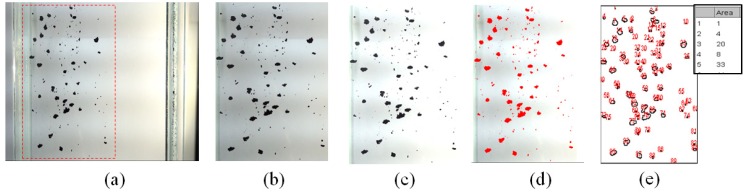
Image processing of CNT particle: (**a**) Original image; (**b**) enlarged image of captured part; (**c**) contrast processed image; (**d**) color thresholding processed image; (**e**) outlined processing.

ImageJ (version 1.50i) [21] software was used for the image processing of photographed particles to obtain the enveloped area of the CNT particles as in Figure 5. The ImageJ proceeds to image processing and image analysis steps [22]. An image of the falling CNT particles to be analyzed is firstly selected as shown in Figure 5b, and a color thresholding process is performed to convert the image into a threshold value which results in coloring the particles (Figure 5d). If some particles are not recognized at this stage, the background and particles are distinguished by the “brightness and contrast” function and the sharpness of the particles is increased by adjusting the brightness as shown in Figure 5c. The area value of the individual particles is obtained through the “analyze particle” function in the image analysis step as shown in Figure 5e. The Heywood diameter (*d_H_*) [23], the equivalent diameter of the circle having the same area as the projected area, was calculated from the area of the CNT particles.

**Figure 6:**
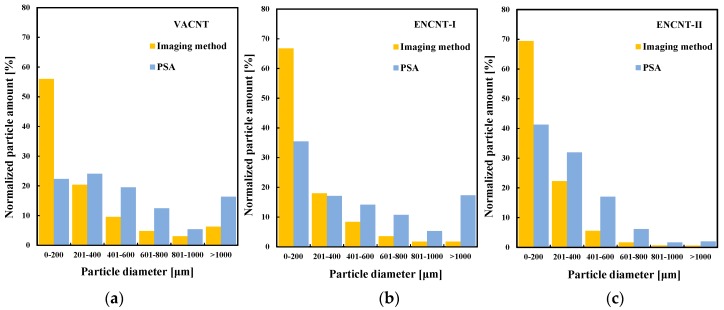
Comparison of particle size distributions from image method and particle size analyzer (**a**–**c**) (PSA: Laser diffraction method).

The obtained particle size from the imaging method shows wide distribution as in Figure 5 and Figure 6. The *d_H_* distributions of the MWCNTs are compared with that obtained from particle size analyzer (PSA: LA-950 V2, Horiba, Kyoto City, Japan) in Figure 6. The *d_H_* distributions have low mode and mean values compared to those by the PSA method. The difference is due to the measurement principle of the PSA using the diffraction of a laser beam by the particles. The prediction of particle size by the PSA strongly depends on both the spatial light scattering patterns and their time dependency as well as on the detectors used for the measurement [24]. Especially, the particle sizing by the laser diffraction is affected by the aggregates shape. For non-spherical particles, the PSA analysis shows the deviating scattering patterns, which may result in a systematic error as if coarse particles were present in the apparent particle size distribution (PSD). [24] Therefore, the particle shape should preferably be incorporated into the PSA analysis model in order to obtain the correct particle size distribution. This is a reason why the equivalent diameter of the aggregated CNTs should be obtained by directly photographing the shape of the CNTs through the proposed imaging method.

Finally, the apparent volume values were calculated assuming that the target particles were spherical based on the Heywood diameter as reported by Wang et al. [22]. Then, the apparent particle density (*ρ_a_*) was obtained by dividing the amount of the injected particles (*M_total_*) by the apparent volume as in Equation (2).

(2) $$\rho_{a}=\;\frac{M_{total}}{\sum_{i}\frac{1}{6}\pi d_{Hi}{}^{3}}$$

#### 2.3.2. Method Comparison

The CNT particle density was measured by a mercury (Hg) intrusion porosimeter (AutoPore V, Micrometrics, US) for a comparison between proposed imaging and previous methods. In the Hg-porosimetry, the volume of mercury which penetrates a porous material is measured with increasing the applied pressure. The apparent volume of the particles can be determined by subtracting the pore volume between particles from the volume of the CNT sample. The particle density of the CNTs can be determined by dividing the weight of the sample by the apparent volume. The details of the measurement and calculation procedures of the Hg-porosimetry can be found elsewhere [16].

For the validation of the proposed method, the particle density of the nonporous PC particles was measured by a pycnometer (ASTM D 854-14 [25]) for obtaining specific gravity and compared with the results by imaging method. The dried and weighed PC particles were prepared for the pycnometry. The particle density was measured using the liquid pycnometer with 95% ethanol as the displacing liquid considering the difference of the specific gravity. The particle density was determined by dividing the weight of the PC particles by the displaced volume [12].

## 3. Results and Discussion

### 3.1. Apparent Density of CNT Particle and Solid Holdup in Loosely Packed Bed

**Figure 7:**
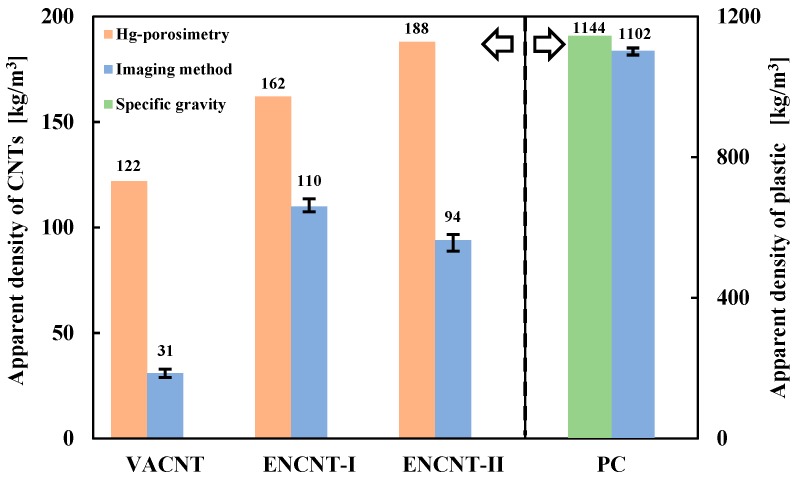
The apparent particle densities by imaging, Hg-porosimety and pycnometer methods.

The apparent density obtained from the imaging method was compared with the value obtained by the Hg-porosimetry method in Figure 7. The apparent density by the imaging method was significantly lower than the value by the Hg-porosimetry method. Interestingly, the difference in the density values was greater for the VACNTs with relatively long nanotubes. The VACNTs are characterized in that the nanotubes grow into long bundles perpendicular to the catalyst surface [5]. The long-grown nanotubes are loosely entangled with each other by the physical twisting and the van der Waals force, resulting in particles or aggregates of a large apparent volume having a large void space inside as in Figure 4 [26]. In this particle structure of high porosity, the Hg-porosimetry method does not sensitively detect the pore volume and apparent volume because the mercury easily penetrates into the void space even at low pressure [27]. Since the ENCNTs have nanotubes of relatively short length compared to the VACNT, the inner void space formed by the interparticle entanglement is small, but the apparent volume is still larger than the true particle volume due to the intraparticle entanglement of the nanotubes. As the result, it shows low apparent densities compared with Hg-porosimetry method. For the validation of the proposed method, the density values of the nonporous PC particles were compared using the imaging method and the pycnometer, and it was confirmed that the imaging method estimated the particle density well within 4%.

**Figure 8:**
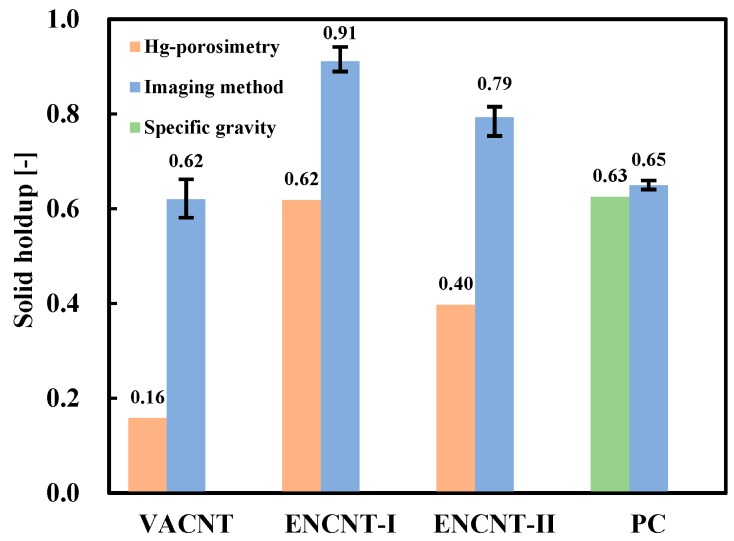
Comparison of solid holdup values of a loosely packed bed.

Figure 8 compares the solid holdup values of the loosely packed bed obtained from the density values (*ρ_a_*) by the imaging method and the Hg-porosimetry method, respectively. The solid holdups of the CNT particles in the bed were calculated by the following Equation (3) after measuring the bulk density (*ρ_b_*) [12].

(3) $$\varepsilon_{s}=\;\frac{\rho_{b}}{\rho_{a}}$$

The bulk density is the density, including the interstitial space between the filled particles, which is the product of the apparent density and the solid holdup [12]. The solid holdups by the imaging method were higher than those from the Hg-porosimetry. As the gas velocity increases in the fixed bed of the CNTs, the phenomenon of gas channeling is observed before complete fluidization of the bed. The channeling occurs because the CNT particles are networked with each other and the gas has a preferential flow by the large drag force against the networked particles [15]. The formation of networked CNT particles means that the enveloped volume directly related to the drag is large. Particularly, the CNT particles contain a lot of fine particles and nanotubes that have been dropped from the particles, and exhibit low bulk density values [28], indicating high values of the solid holdup. 

**Figure 9:**
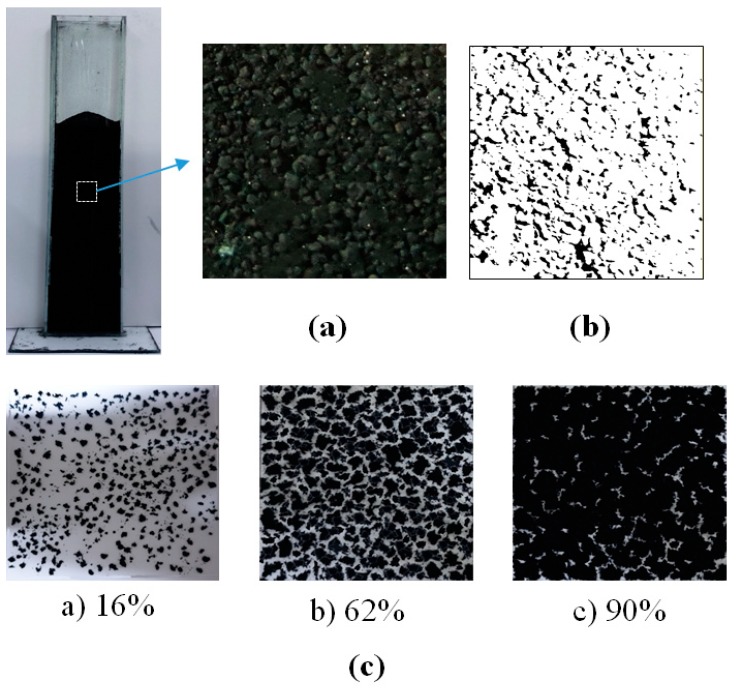
(**a**) Surface photograph of ENCNT-I in loosely packed bed; (**b**) processed image of Figure (**a**); (**c**) two- dimensionally simulated photographs for given solid holdups.

To verify the above results on the methods comparison, a surface photograph of the ENCNT-I particles in the loosely packed bed was obtained and processed with the ImageJ (white color: particles contacting with the surface) as in Figure 9. The processed image (Figure 9b) was compared with the two-dimensionally simulated photographs for given solid holdups as Figure 9c. The simulated images were prepared by scattering the ENCNT-I on a plane according to the surface area ratio corresponding to a given solid holdup. The particles in the ENCNT-I bed are shown to be tightly packed. As a result of the comparison, the area corresponding to the CNT particles observed on the surface is analyzed as 88% of the total area, which is similar to the value from the proposed imaging method (90%). In comparison with the PC particles, the solid holdup by the imaging method was not significantly different from that by a standard test method for measuring bulk density measurement (ASTM D6683-01 [29]).

### 3.2. Solid Holdup in CNT Fluidized Bed

**Figure 10:**
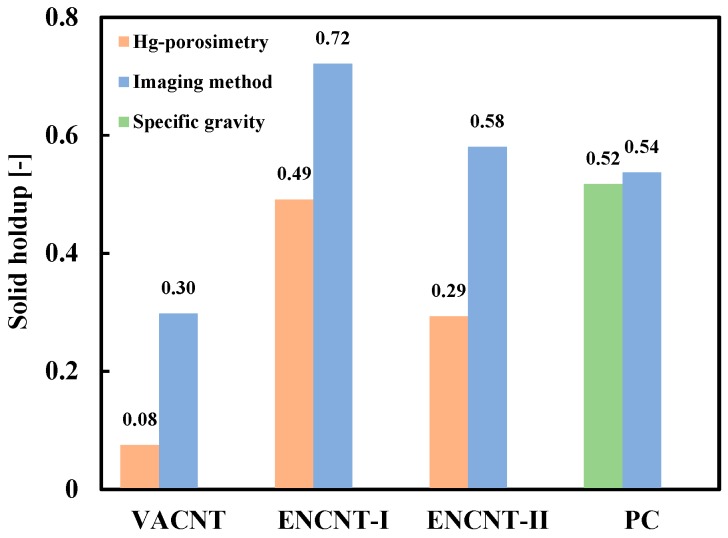
Comparison of solid holdup values at minimum fluidization.

Figure 10 compares the solid holdups at the minimum fluidization, which were based on the imaging and the Hg-porosimetry methods. The solid holdups were calculated by the Equation (1) with the measured pressure drop across the fluidized bed. The solid holdups obtained by the imaging method were higher than those by the Hg-porosimetry, similar to the results in Figure 8. In particular, the solid holdup of the VACNT from the Hg-porosimetry was a very low value of 0.08, which is generally found in the dilute phase of the fast fluidized bed [11] and is hard to occur under the minimum fluidization condition. Interestingly, the solid holdups at the minimum fluidization exhibited relatively low values compared to the loosely packed bed condition. The CNT fines and broken nanotubes are in the void between the CNT particles in the packed bed condition. They are partially fluidized and become adhered to the surface of the CNT particles with increasing gas velocity [16], thereby increasing the voidage of the fluidized bed and exhibiting low solids holdups [8,9]. The solid holdups calculated from the pressure drop of the PC particle at the minimum fluidization conditions showed similar values from both the imaging method and the pycnometer.

### 3.3. Axial Solid Holdup Distribution

**Figure 11:**
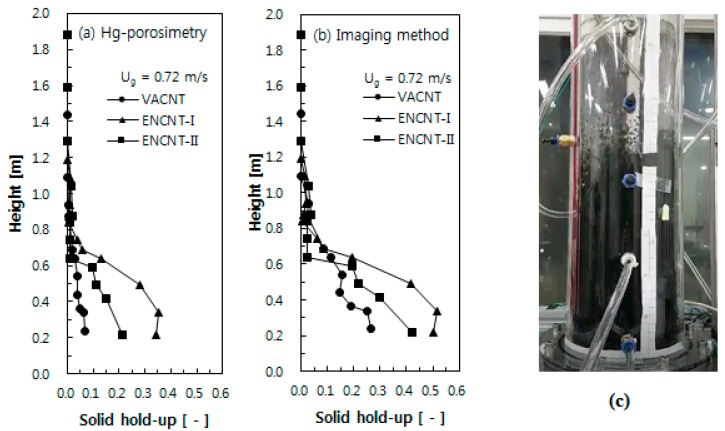
Axial holdup distributions by (**a**) Hg-porosimetry and (**b**) imaging method; (**c**) photograph of of upper part of VACNT fluidized bed at *U_g_* = 0.72 m/s.

Figure 11 shows the axial solid holdup distributions of the CNTs, calculated from different density measurements. The CNT fluidized beds show the solid holdup distributions of a typical bubbling fluidized bed with the lower dense and upper dilute phases after the complete fluidization. The solid holdup distribution calculated by the Hg-porosimetry shows relatively low solid holdups as in Figure 11a, where the VACNTs exhibit very low values of less than 0.07 over the entire bed as if in the dilute phase [11]. However, it can be easily concluded that the fluidized state of the VACNT will have solid holdups above those by the Hg-porosimetry based on the Figure 9c and Figure 11c, confirming once again that the solid holdup distribution predicted by the imaging method can represent well the fluidized bed state of the CNTs. The solid holdups of the VACNT showed lower values than the ENCNTs due to the shape difference of the nanotubes as in Figure 11b. As the gas velocity increases, the long nanotube strands of the VACNT appear to be entangled each other by the physical entanglement and the van der Waals forces, which increases the apparent volume and the voidage between the particles [28].

### 3.4. Comparison of Predicted Sizes of CNTs

Many studies [6,7,30] on the aggregates behavior of nanoparticles and CNT particles reported that the voidage change in the fluidized bed with gas velocity (*U_g_*) can be well expressed by the Richardson–Zaki equation [19] as

(4) $$U_{g}=U_{t}\varepsilon^{n}$$

The CNT mean particle size (*d_a_*) can be estimated by applying the terminal velocity (*U_t_*) obtained from Equation (4) to the Allen equation [31] as Equation (5) when Reynolds number (=*ρ_g_U_t_d_p_*/*μ_g_*) at terminal velocity is greater than 2.0 [6].

(5) $$d_{a}=1.23\frac{U_{t}^{0.875}\rho_{g}^{0.25}\mu_{g}^{0.375}}{{\left(\rho_{p}-\rho_{g}\right)}^{0.625}}$$

**Figure 12:**
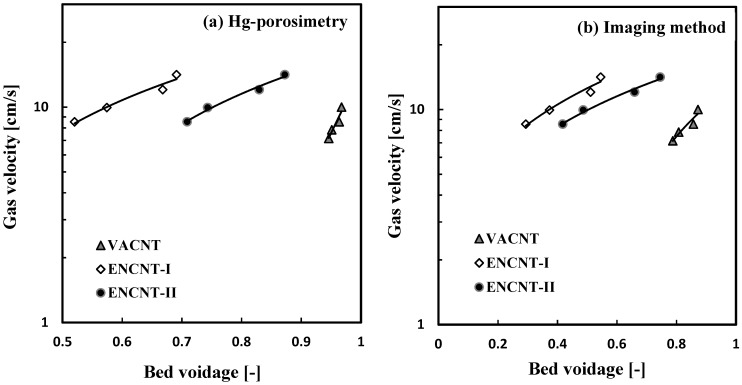
Bed expansion behavior (**a**,**b**) fitted with the Richardson-Zaki equation.

In this study, the measured average particle Heywood diameters (*d_H_*) were compared with the *d_a_* values from Equations (4) and (5), which use the *U_t_* obtained from the voidage (*ε*; *ε* = 1 − *ε_s_*) change and particle densities by the two methods. Voidage changes with *U_g_* are shown in Figure 12. The VACNT and ENCNTs showed different voidage change tendency depending on different flow characteristics by the CNT types. The *U_t_* and n values were obtained by linear regression based on Equation (4) with voidages by imaging method and Hg-porosimetry, respectively. Finally, the measured *d_H_* and the calculated *d_a_* values were compared in Table 2. The particle size by the Hg-porosimetry was very low, because the mercury penetration did not clearly detect the boundary of the enveloped volume [27]. The imaging method was able to analyze the shape of the enveloped volume formed by the entangled nanotubes, and predict diameters similar to the measured size of the CNT particles or aggregates. Finally, it can be seen that the proposed imaging method can obtain reasonable solid holdups in the fluidized bed rather than the Hg-porosimetry.

## 4. Conclusions

A measuring method of the enveloped volume of the entangled CNT particles has been proposed for the information of solid holdups in the CNT fluidized bed. It is characterized by measuring the apparent volume from the CNTs imaging under the free falling condition for the fluidized bed application. The apparent densities and solid holdups of the CNTs and PC particles by the method under various conditions were compared with those by the previous Hg-porosimetry method. The proposed method reflects well the fixed bed and fluidized bed phenomena observed in the experiment with reasonable solid holdups compared with the Hg-porosimetry method. The sizes of CNT particles predicted by the Richardson–Zaki correlation are in good agreement with the measured mean particle diameters.

## Figures and Tables

**Table 1:** Physical properties of particles used in experiment.

Powder	VACNT	ENCNT-I	ENCNT-II	PC
Type	VACNT	ENCNT	ENCNT	Plastic
*d_p_* (μm) ^a^	522	354	291	1140
*ρ_b_* (kg/m^3^)	19	100	75	716

^a^: Measured by particle size analyzer (LA-950 V2, Horiba).

**Table 2:** Comparison of predicted sizes of CNTs by imaging and Hg-porosimetry methods.

-		VACNT	ENCNT-I	ENCNT-II
Measured *d_H_* [μm]		482	308	264
Hg-porosimetry	*n*	2.96	0.74	0.82
	*U_t_* [m/s]	0.15	0.25	0.19
	Predicted *d_a_* [μm]	204	262	190
Imaging method	*n*	13.35	1.62	2.28
	*U_t_* [m/s]	0.15	0.21	0.18
	Predicted *d_a_* [μm]	474	292	282
